# Supplementary material for: How well did experts and laypeople forecast the size of the COVID-19 pandemic?
Source: PLoS One. 2021 May 5;16(5):e0250935. doi: 10.1371/journal.pone.0250935 (PMC8099086; doi:10.1371/journal.pone.0250935)
Supplement: S1 Appendix — Expert and nonexpert questionnaire items. (DOCX) [file pone.0250935.s001.docx]

**S1 Appendix**

**Questionnaire items**

In which country do you currently live?

________________________________________________

***[The following question appeared only in the expert survey distributed on social media. Although the recruiting advert specified that we were looking for statisticians, modelers, epidemiologists, virologists, or clinicians to complete the survey, we nevertheless included this question and excluded data from individuals who only marked “Other” and did not mark any of the other options.]***

What is your occupation or specialty? You may select more than one if multiple options apply.

- Epidemiologist (1)
- Statistician (2)
- Mathematical modeller (3)
- Virologist (4)
- Clinician (5)
- Other (please specify) (6) ________________________________________________

Now we're going to ask you about numbers related to coronavirus/COVID-19. The answer to each question is a number. We will ask you what you think the true answer is.

 We will then also ask you for a range around that answer that you're 75% confident contains the true answer.

 For example: If you think the number of balls in a bag is 10, but think it might be as high as 15 and as low as 5, you would enter '10' as the answer but then for '5 - 15' for the range.
   We're interested in what you think the 'true answer' is, but we're also very interested in how sure you are about that answer - that's why we're asking you to give us a range.
   Please choose a range in such a way that you think there's about a 75% chance that the real-world answer will fall between your lower and higher number.

How many people **in the country you're living in** do you think will have **died** from COVID-19 by December 31st 2020?

________________________________________________________________

Please type the range where you are 75% confident that the true answer will turn out to fall between the two numbers you've indicated.

 Please type the lower number and the higher number with a dash between them.

________________________________________________________________

How many people **in the country you're living in** do you think will have been **infected** with COVID-19 by December 31st 2020?

________________________________________________________________

Please type the range where you are 75% confident that the true answer will turn out to fall between the two numbers you've indicated.

Please type the lower number and the higher number with a dash between them.

________________________________________________________________

**Out of every 1000 people who will have been infected** by the virus **worldwide**, how many do you think will have died by December 31st 2020 as a result?

________________________________________________________________

Please type the range where you are 75% confident that the true answer will turn out to fall between the two numbers you've indicated.

Please type the lower number and the higher number with a dash between them.

________________________________________________________________

**Out of every 1000 people who will have been infected** by the virus **in the country you're living in**, how many do you think will have died by December 31st 2020 as a result?

________________________________________________________________

Please type the range where you are 75% confident that the true answer will turn out to fall between the two numbers you've indicated.

Please type the lower number and the higher number with a dash between them.

________________________________________________________________

The adaptive version of the Berlin Numeracy Test was only included in the nonexpert survey and was administered only after the questions above had been answered. The test items and scoring instructions are available in Appendix 2 of:

Cokely ET, Galesic M, Schulz E, Ghazal S, Garcia-Retamero R. Measuring Risk Literacy: The Berlin Numeracy Test. Judgm Decis Mak. 2012;7(1):25–47.
