# Supplementary material for: How well did experts and laypeople forecast the size of the COVID-19 pandemic?
Source: PLoS One. 2021 May 5;16(5):e0250935. doi: 10.1371/journal.pone.0250935 (PMC8099086; doi:10.1371/journal.pone.0250935)
Supplement: Attachment — Submitted filename: Response to reviewers.docx [file pone.0250935.s002.docx]

This is a very interesting manuscript addressing a timely question of disease forecasting. Though reviewers mainly pointed out some minor issues that need to be addressed, providing more clarification on the use of terms 'expert' and 'non-expert' will greatly improve the quality and potential impact of the study. To this effect, I fully agree with Reviewer #1 suggestions, and strongly encourage you to address them thoroughly.

**Thank you for the kind words. We have undertaken substantial revisions to address both reviewers’ suggestions, described in detail below, and feel that the paper is much improved as a result.**

Reviewer #1: Summary

While expert opinions have exerted a large influence on public policy during the Covid-19 pandemic, relatively little research has been done that examines the accuracy of such expert predictions.

To fill that gap, the paper shows the results of a survey done in April on experts and non-experts who were asked to submit a prediction for four different covid-related targets. The paper makes a compelling case that experts (and to an even greater extent non-experts) have a tendency to be overly certain in their estimates and underestimate the possibility of extreme events. It is interesting to see that this tendency was different between experts and non-experts. It is also interesting that off-the-cuff predictions, on average, probably are not helpful to inform public policy.

Major issues

The idea of comparing experts and non-experts is in principle very interesting - especially given the importance of expert opinion for shaping public policy. One factor that makes studying this subject difficult, however, is the immense variability hidden behind the terms 'expert' and 'non-expert' that is (naturally) hard to capture. For example, a scientific policy advisor may be a quite different type of expert (with potentially much better forecasting skills) than someone who simply has a statistics background. Similarly, a layperson putting a lot amount of effort into forecasting questions on Metaculus, or someone who is a Superforecaster (see <https://goodjudgment.com/>) may make forecasts very differently than the average person who would participate in a study through Prolific Academic or Respondi.

One way in which I especially fear study participants may differ from either 'experts' who inform policy or voice predictions publicly, or 'non-experts' who make public predictions is 'skin in the game' and the incentive structure they are faced with. Experts who make public predictions and who take responsibility may have very strong incentives to be as correct as possible (and possibly as cautious). Even if they make off-the-cuff prediction, they will probably be aware of public scrutiny. Similarly, laypeople who predict to earn money or to rank highly on a public leaderboard also have an incentive to be right. This likely greatly enhances predictive accuracy (see e.g. literature on Superforecasters). Experts surveyed in the study however, as I understood, had no real incentive to be right (except for the fact that they were recruited as 'experts' and therefore possibly felt some psychological pressure to live up to that standard). Non-experts presumably had no incentive at all to be right and maybe even had a financial incentive (in terms of their hourly rate) to complete the online survey quickly. The authors briefly mention how some people on Metaculus may be outperforming experts consistently, but unfortunately do not address this further or contextualise it in light of their research.

I feel it would be helpful if the authors were more explicit on these limitations or address them otherwise. They do state unequivocally how they recruited their sample of 'experts' and 'non-experts', but could be clearer on the implications of that selection process and the study design. It would help to expand on how comparable the 'experts' in the study are to the 'experts' that actually do inform public policy. In the paper the same term 'expert' is used in two ways: First in the introduction and conclusion that talks about how important expert opinion is for public policy, and secondly to denote the group of people in the study with a background in certain fields. To the reader, this implies some comparability. I think it needs more explanation to argue that forecasts from the former group are actually comparable to predictions from the latter - or an explicit statement that they may not be. One option could be to call them 'study participants with a background in subject relevant fields' (or probably a catchier version of that) in the paper. Similarly, I think it should be addressed that the group of laypeople in the study may not be able to represent all kinds of 'non-experts' predictions, or even the ones we might care about most (e.g. Metaculus, Predictit etc.). That being said I do find it intuitively plausible that all experts, including those making public predictions have a tendency to be overconfident, even if they are incentivised to be right. I just believe this link needs more supporting evidence. One way to do show this could be to check for explicit predictions made by experts around the same time and see whether they are comparable with what the study participants predicted.

**Thank you – these are important points, and we have undertaken substantial revisions to address them. First, we have made our operational definition of ‘experts’ more explicit in the abstract and introduction, and highlighted the distinction between the ‘experts’ in our study as opposed to experts who most directly inform public policy (e.g., scientific advisory committees, scientists participating in science policy initiatives, etc.) in the introduction.**

**More substantively, the discussion now notes the heterogeneity among experts and the conditions under which they make forecasts as a key limitation with respect to what this study can tell us about expert predictions in the real world, and acknowledges that it is an open question to what degree any findings about off-the-cuff predictions of the ‘experts’ recruited in our study might generalize to predictions made by those experts who have an explicit mandate to produce forecasts for policymakers. It also now goes into substantial detail regarding the causes of this heterogeneity, including incentive structures. There is also a long tradition of research into expert overconfidence, which we also now discuss in some detail. Some of this research suggests we have reason to believe our results may generalize to experts making public predictions, at least to some degree. We also completely agree that forecasting experience and the incentive structure of the social environment in which the forecasts are made will have an effect (for experts and nonexperts alike), and go into further detail than we had previously on the generally impressive performance of some groups of individuals who are not subject-matter experts, such as the Good Judgment Project and the predictors at Metaculus (whose performance is clearly far better than that of our ‘nonexperts’). Ultimately, we conclude that “our findings of overconfidence in lay and expert COVID-19 predictions are consistent with what would be expected from literature on predictions and judgments in other domains. They seem likely to generalize to common real-world contexts in which everyday people encounter expert or nonexpert predictions (e.g., within traditional or social media), but not necessarily to all such contexts. They also may not generalize to contexts where accuracy and good calibration are strongly incentivized, or in which individuals receive systematic training or practice in forecasting with regular feedback.” Finally, we discuss data from other expert surveys, which illustrate both a few examples of clear expert overconfidence with respect to short-term COVID-19 predictions, but also some more appropriately humble expert predictions, as well as COVID-19 predictions made by predictors on Metaculus and The Good Judgment project in May. The U.S.-centric nature of these surveys means they cannot be directly compared to our numbers, but they illustrate some of the points made previously in the discussion: 1) we might reasonably expect the overconfidence observed in our survey to generalize to some, but not all, other contexts; and 2) nonexperts practiced in participating on forecasting platforms, such as the Good Judgment Project and Metaculus, do not seem to be doing particularly badly – so we should not expect our findings of poorer ‘nonexpert’ performance to extend to these kinds of contexts.**

Minor issues

. line 66 page 3 the reporting of the Ebola study is worded slightly confusingly. What is 4 of 7?

**This has been reworded for clarity.**

- line 123 I believe it should be 'divided by 1,000' instead of multiplied by 1,000

**As the question (question 4) was “Out of every 1000 people who will have been infected by the virus in the country you're living in, how many do you think will have died by December 31st 2020 as a result?”, the infection fatality rate of 1.18% (.0118) needed to be multiplied by 1000 to get the answer.**

- In general, I feel it is somewhat unfortunate that for 3 out of 4 questions, there is no actual "truth data", but instead only estimates. This should ideally be thought of beforehand for future studies

**This was partly due to a prediction failure of our own: In March 2020 when the study was designed, there was much focus on assessing the disease’s infection fatality rate, as well as concern about the difficulty of assessing true death counts on a national level and whether governments would be transparent about these counts. We therefore assumed that there would be more consensus around infection fatality rates by the end of the year than there would be around death counts, and imagined that we would not need to rely on estimates for questions 3 and 4. That being said, the differences between expert and nonexpert estimates were so stark that any reasonable estimates show the experts to have been better predictors. In retrospect, we should have given more thought beforehand to exactly which sources we planned to treat as authoritative, and asked the questions accordingly.**

- Specifically for question 3 (lines 123ff) it is unclear to me what was multiplied with what to obtain death numbers. I'm also not quite clear why the authors did not simply take publicly reported death numbers?

**We have modified the paragraph to give more detail on the calculation. Because question 3 asked “Out of every 1000 people who will have been infected by the virus worldwide, how many do you think will have died by December 31st 2020 as a result?”, it is effectively asking for the worldwide infection fatality rate (multiplied by 1000). Without knowing how many people were infected by the virus, the worldwide death count is of little help here.**

**As for the questions we chose to ask, we absolutely should have included a question simply asking participants to predict the worldwide death count, and we regret not including such a question!**

- Regarding the construction of linear opinion pools: the authors could maybe be clearer on

a) why they chose to construct distributions for every individual expert at all. Was this just for the purpose of scoring, or was this mainly to construct an overall aggregate distribution?

**This was done to enable the calculation of the CRPS, and also to construct an overall aggregate distribution for visualization purposes. We have now clarified this in the section describing the construction of the linear opinion pools.**

b) how their choice of restricting the distribution with 'width/2' was motivated?

**Having distributed 75% of the probability mass within the range provided by the participant, we were left with the question of how to distribute the remaining 25%. Although we were very happy to make simplifying assumptions, distributing the remaining 25% uniformly among all other possible values seemed perhaps too simple, as this would imply that an individual’s estimated probability of (say) a number of deaths just outside of their ‘75% confidence range’ was equal to their estimated probability of 0 deaths, and of 67 million deaths. We therefore made the decision to restrict the distribution. If we were to assume that the participant’s range corresponds to the “middle 75%” (i.e., the 12.5th to 87.5th percentiles) of a Gaussian distribution, two times this width (i.e., extending it to the left and to the right by width/2) would span from the 1st percentile to the 99th percentile. It therefore seems likely that an individual who is truly 75% confident that a value will fall between *lower* and *upper* would be extremely surprised if the value was less than *lower – (width/2)* or higher than *upper + (width/2)*. Restricting the distributions within these bounds therefore seemed justified. We have now noted this in the paper.**

**There are of course other ways that the distributions could have been constructed for each participant, for example, generating Gaussian distributions such that 75% of the probability mass fell within the participant’s interval, and then skewing them so that their medians (or some other measure of central tendency) became equal to the participants’ point estimates - of course, even this approach would have required making different arbitrary decisions and assumptions. Again, though, the differences between groups were so stark that the specific approach used should not affect the fundamental findings.**

c) how exactly they aggregated individual expert distributions to one distribution. Did they combine the CDFs horizontally, i.e. use a quantile average, or vertically, i.e. construct a mixture distribution?

**Probability distributions for different individuals were combined with simple averaging of the probabilities (i.e. ‘vertical’ combination). We have now noted this in the paper.**

- Regarding the scoring of forecasts it could be made clearer why the authors used the crps instead of weighted interval score (e.g. Bracher et al. 2021 <https://doi.org/10.1371/journal.pcbi.1008618>). It seems much more intuitive to calculate the wis for every individual expert and average it for every subgroup (experts, non-experts etc) the authors are interested. This is much easier than constructing a full predictive distribution and using the crps to score it. A software implementation of the WIS is for example available in the scoringutils package (<https://github.com/epiforecasts/scoringutils> - Disclaimer: I'm an author of that package).

**This would have been a smart move and we will keep it in mind for the future! We have added the statement: “An alternative, perhaps more straightforward approach would have been to use the weighted interval score (Bracher et al. 2021), which approximates the continuous ranked probability score and does not require the construction of a full predictive distribution.”**

- Regarding the assessment in terms of relative scores in lines 176ff: I am unsure whether the relative assessment is actually necessary, as the main message is already clear in terms of absolute differences and so a relative assessment doesn't seem to add much.

**We felt that for some readers, the mean relative error would likely feel more informative than the mean absolute error. (Knowing that an estimate is off by some particular value feels very different if this estimate is only off by 1% versus if it is off by 300%). Having reported a measure of mean relative error in Table 1, we felt we should at least devote a sentence or few explaining what that measure actually represents. We agree that the statistical tests on mean relative error may be superfluous given that we have already reported similar tests on mean absolute error, but as they take up only two short sentences we saw no harm in leaving them in.**

- stylewise, the reporting of the numerical results in the sections "Calibration" and "Continuos ranked probabilty" is somewhat hard to follow as there are a lot of numbers, maybe a table would be better suited to present the different p-values and tests performed? Especially in the section on the crps, the authors could be clearer on what numbers they report (for example, I'm not sure what the negative numbers mean).

**We have now reported these numerical results in tables rather than in the main text, and have clarified what tests were performed and why. The negative t-statistics merely reflected the order that the variables were entered into the t-tests; as these are two-sided tests, the order of entry and the sign are irrelevant. We agree that reporting negative t-statistics in this context is confusing, and we have now flipped the order that we entered the variables, flipping the sign to positive.**

- A minor point regarding the assessment of calibation in lines 188ff: From the text I understand that the authors assume that a forecaster whose prediction intervals cover more of the true values are better calibrated. While this seems true in the case where all participants are overly confident in their predictions, it doesn't hold in general: a forecaster who covers 100% of the observed values with his prediction intervals (instead of the 75% aimed for) is not necessarily better calibrated than one who is right 50% of the time.

**This is an excellent point. We have reworked the section on calibration accordingly.**

Reviewer #2: The paper analyzes the precision of forecast regarding the magnitude of the COVID-19 pandemic by experts and non-experts. They compare the forecast in spring 2020 at the initial stage of the pandemic with the outcomes in December in the same year. They found that experts perform better in both accuracy of prediction and accuracy of levels of confidence than non-experts. The research addresses an important policy-relevant question.

Major comments.

1. They compare raw data across groups. However, individual characteristics, such as gender or age, may explain some of the observed difference. Please compare the distribution (or mean) of individual characteristics across the three groups. I suspect the distribution is not necessarily similar given their sampling scheme. If it is the case, is there any way to control for the difference in some of the analyses?

**There are indeed some differences between groups on individual characteristics, but they do not explain the differences in accuracy and calibration between experts and nonexperts. Experts had a mean age of 42.3 (95% CI 40.0 - 44.7), slightly younger than nonexperts’ mean age of 45.3 (95% CI 44.6 - 46.1). Experts were also 75% (66% - 83%) male; this proportion was higher than either the high-numeracy nonexpert group -- 51% (47% - 56%) male -- or the group of all nonexperts, 48% (46% - 51%) male. However, by including all of these variables together in the same regression model, we can see that these differences do not seem to explain the differences in expert vs. nonexpert calibration and accuracy. When regressing calibration on gender, age, and expert/nonexpert status, expert status (ꞵ=1.3) and male gender (ꞵ=0.2) were both significantly associated with better calibration. When regressing rank-transformed error on gender, age, and expert/nonexpert status, expert status was associated with lower error for each of the four prediction questions, with betas for expert status being 2 to 16 times the magnitude of the corresponding beta for gender, depending on the question. Older age was associated with (lower) error for question 4 only.**

**Numeracy could not be included in these regressions as we did not require experts to complete the Berlin numeracy test. However, if these regressions are repeated but with nonexperts restricted to the subset of high-numeracy nonexperts, expert status continues to significantly predict calibration, and also continues to significantly predict rank-transformed error for each of the four questions with the exception of question 2.**

**Restricting the dataset to only nonexperts allows numeracy to be included as a predictor along with age and gender. Higher numeracy was significantly associated with better calibration, as well as with lower error on each prediction question. Male gender remained associated with better calibration, and also remained associated with lower error on three of four prediction questions, but the beta for gender had lower magnitude than the beta for numeracy in each case. As before, age was associated with error for question 4 only.**

**Overall, these follow-up analyses bolster our confidence that differences between experts and nonexperts, and differences between high-numeracy nonexperts vs. all nonexperts, are not merely due to confounds with age or gender. We have added these analyses to the results and noted them in the Discussion.**

Comments.

2. P4. L86.There are two ways to survey non-experts: Prolific Academic and Respondi.com. What’s the reason for using two ways for survey? Is there any difference in respondents’ characteristics and/or responses? Please explain.

**At the time this experiment was run, our centre was in the habit of using Prolific as well as Respondi for all surveys to learn more about the differences between participants on each platform, and to verify that findings in other studies in the same battery were not specific to a single platform. Given our proportional quota sampling procedure, there were unsurprisingly no significant differences in age or in the proportion of male/female participants between the two platforms. However, participants from Prolific had higher numeracy scores (mean 2.2, 95% CI 2.1 - 2.3) than those from Respondi (mean 1.79, 95% CI 1.73 - 1.85). Participants from Respondi likewise had poorer calibration and poorer accuracy on each question. It is unclear exactly what accounts for the difference between participants from Respondi and Prolific. Prolific historically has been marketed towards more scientifically literate individuals (it originally marketed itself as “the world's largest crowdsourcing community of people who love science”), so there may be differences in the interests and backgrounds of participants on the two platforms.**

**Nevertheless, these differences do not seem to make a difference to our findings. For example, repeating the regressions previously described but restricting the nonexpert dataset to only participants who were recruited on the Prolific platform, we again find that expert status continues to significantly predict (better) calibration, and also continues to significantly predict (lower) rank-transformed error for each of the four questions, with the exception of question 2, where there is no association. (These are exactly the same results we found when we restricted the regressions to the subset of high-numeracy nonexperts). If the nonexpert dataset is restricted to only participants recruited on Respondi, expert status significantly predicts (better) calibration, and (lower) rank-transformed error for each of the four questions.**

3. P4. L84. How do they pick up the particular value of 75% for calibration? Is this the common number used in literature?

**One reason was that we wanted a value that would make good calibration straightforward to identify: given that four questions were asked, the number of outcomes falling within an optimally calibrated individual’s ‘75% confidence range’ has an expected value of exactly 3. That said, it also has some precedent in the literature, though other values are commonly used as well. Ruscio (2000) sums up why a 75% confidence interval is reasonable: “First, this value was relatively easy for participants to understand. They simply needed to construct intervals so that they were likely to be correct three-fourths of the time. Second, because variation around 75% was possible, this value was sensitive to both overconfidence and underconfidence. Third, the 75% value apportioned greater sensitivity to discriminating variations in overconfidence than in underconfidence. Given that underconfidence is relatively rare, this seemed appropriate.” We have added a note to our paper highlighting this second point.**

**Ruscio, J. (2000). The role of complex thought in clinical prediction: Social accountability and the need for cognition. *Journal of Consulting and Clinical Psychology, 68*(1), 145.**

4. P9. L162. Please explain what CRPS shows in the method. It seems it analyzes the accuracy of forecasting, but I would like to know more details on why these analyses are necessary.

**The continuous ranked probability score is one of the most common ways of comparing the performance of different probabilistic forecasts of the same outcomes. In effect, it is a measure of accuracy that produces the best (i.e., lowest) scores for accurate judgments about which the participants are highly certain. Although we computed the CRPS of the aggregated consensus distributions, if we were to compute it for an individual forecast (after transforming that forecast to a probability distribution in the manner described in our paper), it would most reward participants with relatively narrow 75% ranges that also contained the correct outcome; it would give poorer scores to participants whose 75% ranges contained the correct outcome, but were somewhat wider; and would give especially poor scores to participants with narrow 75% ranges that were not anywhere close to the correct outcome. It is useful in this study as it serves as a measure of accuracy that can be applied to a consensus distribution constructed from participants’ 75% ranges, rather than from their point estimates. We have now explained this in the paper.**

5. P10. L211. Please explain for what hypothesis test the t statistics are used in CRPS analyses.

**Yes, we had not made this clear - it has now been clarified in the corresponding section.**

6. Discussion. The next obvious question is where the difference between experts and non-experts come from. Is it because of their scientific knowledge, difference in information source or something else? I understand this is not the question the current study could address from the survey, but any discussion would be informative for readers.

**This is a good question, although we have little to go on but speculation. ‘Relevant subject-matter expertise’ typically includes knowledge of what real-world data sources contain the most reliable information; for example, it seems plausible that our ‘experts’ did well on questions 3 and 4 simply because they were aware of preliminary estimates of the infection fatality rate, whereas our nonexperts may not have been. Individuals with professional backgrounds in statistics, mathematical modelling, and epidemiology also seem likelier to have had a greater familiarity with the raw data around COVID-19 infections and death rates, a firmer grounding in how to interpret that data, and the high levels of uncertainty associated with epidemiological forecasts in general, which could all account for the difference. We now make note of this in the discussion.**

7. Discussion. Does this study have any implication for the context outside of non-infectious diseases? In other words, to what extent this is related to the non-linear dynamic nature of infectious diseases?

**It is certainly plausible that all predictors were as inaccurate as they were (though the experts slightly less so) in part because the non-linear dynamic nature of infectious diseases makes possible futures especially uncertain: small initial differences in infection parameters can lead to big differences in outcomes with time. It seems plausible that this also makes it challenging to estimate one’s own level of certainty, as we have now noted in the discussion. However, overconfidence in forecasts is not necessarily restricted to experts or to epidemiological forecasts, as it has been shown in a variety of other fields; we have now cited extensive literature in the Discussion that makes this point.**

**Thanks to the editor and to all reviewers for these points; we certainly feel that the suggestions and changes have improved the paper and made it more useful to readers.**
